# Axillofemoral bypass in elderly patients with local anesthesia: an alternative route to less risk

**DOI:** 10.1186/1471-2482-13-S1-A6

**Published:** 2013-09-16

**Authors:** Enrico Cappello, Mario Di Lorenzo, Enrico Cutillo, Elio Franco

**Affiliations:** 1 Unit of Vascular Surgery, Rummo Hospital, Benevento, Italy

## Background

The evolution of medical and minimally invasive techniques have led to improved outcomes in limb salvage in elderly patients. In particular, has improved the overall survival of the patients and life expectancy.

The patient with major amputation have high rates of mortality and morbidity and health care cost with a considerable impact on society.

The work presented here concerns elderly patients with aorto iliac occlusive disease and limb ischemia with and without ulcers.

Today revascularisation technique of choice, in these patients, is the endovascular recanalization. In some cases, this technique is not feasible due to the impossibility to cross the occlusions (about 10% of the procedures). The aorto- femoral bypass "anatomical" is to be preferred to the “extranatomical” because it has best primary and secondary patency rates [1-4].

In patients with high surgical risk and big morbility is not advisable to realize the aorto-femoral bypass for the high operative risk. In these patients, the only alternative to major amputation is the bypass extranatomical (femoro-femoral or axillofemoral).

We prefer to use a spinal anesthesia with high back puncture for femoral access associated with a local anesthetic for axillary access. This approach reduces both the risk: free surgical opening of the abdomen and general anesthesia.

The aim of treatment is limb salvage. In fact, at times, it is observed that patients do not return to critical ischemic phase even if the graft is occluded.

In patients treated the intervention is well accepted. The patient does not require post-operative intensive care, begin to ambulate early and requires low doses of painkiller. Patients who may benefit from this treatment are those with too much risk for aortic revascularization. We prefer to avoid the femoro-femoral bypass because of controlateral limb have latent ischemic arterial disease that may worsen afther robbery blood graft.

## Materials and methods

We included 15 patients treated between 2005 and 2012. They had a critical lower limb ischemia or skin ulcers (stage III or greater Fontaine classification). The average age was 84 years. Patients had significant risk factors: ischemic heart disease, lung disease, respiratory failure, heart failure, diabetes mellitus, moderate-severe hypertension, cerebrovascular disease. The operative risk was classified according to the ASA classification by anesthesiologists and all patients were ASA III or higher. Before surgery was planned a meeting between surgeons, anesthetists and cardiologists.

All patients underwent a CT angiography and lower extremity angiography to evaluate the landing zone of the bypass between common and profunda femoral artery.

We use always a PTFE prosthesis heparin linked (Gore-propaten ®) 8mm diameter.

A recent study has shown that the prosthesis 8mm diameter has patency rates best of 6mm, due to an increase wall stress in the prosthesis with smaller diameter with intimal hyperplasia [5].

Anesthesia was performed with a lumbar high puncture supplemented by local infiltration of 2% lidocaine and 0.25% bupivacaine with 1:1 ratio for axillary access and thoracic subcutaneous tunneling. A mild sedation with midaziolam allowed to keep the patient liking. The mean operative time was 130 minutes, and the maximum dose of anesthetic was 100ml.

The surgery was performed by 4 operators 2 for femoral access and 2 for axillar to reduce the time.The follow-up was performed at 1,3,6,12 months evaluating the patency of the graf, the mortality and limb salvage.

## Results

All patients passed the intervention with intra and perioperative mortality rate of 0%. The rate of limb salvage at 30 days was 100%. At 12 months, 4 grafts were occluded and only 1 patient required a major amputation for occlusion of the graft and the worsening of the clinical presentation with failure rates of 26% (occlusion of the graft without clinical ischemia) and 6% (major amputation for procedure failure) respectively.

In 9 cases it was necessary to perform an anastomosis on proximal profunda femoral artery and in 6 cases on distal. None of the patients had infections of prosthetic bypass or anastomotic aneurysms.

The most obvious result was the immediate and complete resolution of pain foot and clinical improvement. In 5 cases, at a distance of 30 days, it was performed a partial amputation of the foot for the removal of gangrene with complete recovery in about 6 months.

## Conclusions

The axillofemoral bypass is the last viable for limb salvage in high-risk patient with aorto iliac disease. Reduce the risk with intraoperative local anesthesia has, in our view, represented a further indisputable advantage for the elderly patient. In fact, these patients have associated risks of general anesthesia with its need post-operative intensive treatments also prolonged. This procedure reduces post-operative cardiac and pulmonary complications. It allows rapid mobilization of the patient and an immediate return to self-employment life management. Patients accepted the intervention without major trauma and, in particular, the disappearance of chronic pain in the foot provides an improvement in the quality of life enjoyed immediately after the surgery.

With this procedure we do not think that we have solved all the problems of the elderly patient with critical limb ischemia, but use it with local anesthesia has made us re-evaluate the role of “extra-anatomical” revascularization. Today more than ever, patients have a life expectancy getting longer and longer and are presented to the vascular surgeon with increasingly complex frameworks that require a continuous search for new strategies and techniques designed to reduce the risk factor in patients with high surgical risk.
